# Optimizing the Interpretation of Cardiopulmonary Exercise Testing in Endurance Athletes: Precision Approach for Health and Performance

**DOI:** 10.1155/tsm2/5904935

**Published:** 2025-08-20

**Authors:** Tomasz Kowalski, Przemysław Kasiak, Tomasz Chomiuk, Artur Mamcarz, Daniel Śliż

**Affiliations:** ^1^Department of Physiology, Institute of Sport, National Research Institute, Warsaw 01-982, Poland; ^2^3^rd^ Department of Internal Medicine and Cardiology, Medical University of Warsaw, Warsaw 02-091, Poland

**Keywords:** cardiopulmonary exercise testing, cardiorespiratory fitness, endurance athletes, exercise physiology, sports cardiology

## Abstract

The present review summarizes findings from the NOODLE (“predictioN mOdels fOr enDurance athLetEs”) study. The research aimed to refine variables obtained during cardiopulmonary exercise testing (CPET) in a large cohort of highly trained endurance athletes by adjusting general reference values and predictive equations to better reflect the unique physiological profiles of this population. Ventilatory efficiency, oxygen uptake efficiency slope, oxygen uptake efficiency plateau, and peak oxygen pulse were analyzed, as they were recently applied in various models concerning risk stratification and treatment optimization. As more people engage in endurance sports, tailored CPET assessments are crucial for accurate performance evaluation and health monitoring. By characterizing differences between general formulas and those suited for endurance athletes, we offered improved tools for optimizing training and ensuring athlete safety. The findings are in line with the existing trend of precision medicine that tailors diagnostics, treatments, and interventions to individual patients' characteristics. Moreover, we review the recent advances from widely applied CPET-obtained indices, such as maximum oxygen uptake, maximum heart rate, and breathing reserve. We also gave the recommendation for a comprehensive CPET assessment based on the relationships between all of the variables.

## 1. Introduction

Cardiopulmonary exercise testing (CPET) plays a crucial role in assessing the health and performance of endurance athletes, offering insights into both cardiovascular and pulmonary function [1]. By measuring key parameters, CPET provides a detailed profile of an athlete's aerobic capacity, respiratory efficiency, and metabolic responses to exercise and may contribute to medical diagnosis and risk stratification, among many [2]. In athletic settings, the effort is typically continued until exhaustion to identify maximum variables such as oxygen uptake, heart rate, ventilation, and carbon dioxide production. Moreover, submaximal indices like ventilatory or lactate thresholds, as well as movement economy, are often defined as common performance determinants in endurance sports [3]. CPET results are usually interpreted in the context of reference values and predictive equations. They play a vital role in medicine and diagnostics, serving as benchmarks to interpret clinical data and assess individual health status. By comparing the obtained results to the established norms, deviations that may indicate underlying conditions or risks should be detected. Such an approach allows for precise evaluation of physiological function and helps to guide diagnosis, treatment, and monitoring of various populations [1, 4]. For endurance athletes, reference values and predictive equations specifically tailored to their cardiorespiratory profile seem essential. Traditional CPET reference values may not apply because endurance athletes exhibit different characteristics compared to the general population [2]. The values are typically higher, especially for gold-standard measurement, i.e., peak oxygen uptake (VO_2_peak), as presented in Table 1. When comparing reference values for CPET for general and athletic populations, the VO_2_peak is higher among athletes in each case [5, 6]. Possible reasons may include exercise-induced electrical and structural cardiac remodeling, as well as specific functional adaptations [7–9]. Therefore, differentiation between normal physiological adaptations and potential pathology requires careful consideration [10]. Equations specifically developed for endurance athletes allow for a more accurate interpretation of their CPET results, ensuring that performance and health risks are appropriately assessed. Moreover, CPET application varies between particular sports disciplines. CPET could be used both for training and diagnostic purposes [11]. Most often, CPET is conducted up to the maximal exertion [12]. However, in several parts of the sports season for some disciplines (e.g., triathlon), intensity of the CPET could be downgraded to the submaximal to not overtrain the athlete [13]. It is especially useful in the final parts of the sport's season when exertion reaches its peak. CPET is usually performed on the treadmill or cycle ergometry [4]. For some disciplines like watersports, the most mirroring modality will be rowing ergometry [14]. To sum up, it is well recommended that the modality and intensity of CPET should be considered when interpreting the results.

Noteworthily, in recent decades, the number of endurance athletes has significantly increased [15–17]. Many individuals participate in endurance sports, both professionally and as amateurs, while undertaking significant training loads [18]. Although physical activity is associated with multiple health benefits and is an undisputed positive lifestyle factor, it may also bring noteworthy risks [19, 20]. Especially many amateurs perform strenuous training with limited medical screening and supervision, which may result in severe health issues, possibly leading to sudden cardiac fatalities [21]. Interestingly, only one year of endurance training may result in morphological adaptations like those in elite athletes [22]. As a result, there is a growing need for tailored CPET evaluations to support clinicians and coaches in monitoring an athlete's health, detecting early signs of overtraining, and optimizing training regimens to improve performance safely. Moreover, novel CPET-obtained variables have recently been suggested for inclusion in risk stratification and treatment optimization processes [23–26]. However, they were typically assessed in patients or the general population, and tools allowing for application in endurance athletes are lacking. Consequently, we developed the NOODLE (“predictioN mOdels fOr enDurance athLetEs”) study, which resulted in a series of articles addressing the possible differences in reference values and predictive equations between the general population and endurance athletes [27–30].

In our research, we investigated whether there are significant differences between the well-established CPET-obtained variables used for the general population and those that apply specifically to endurance athletes. As the type of CPET impacts the analysis of results, we additionally stratified the interpretation by modality [11]. We hypothesized that general predictions and equations might not accurately reflect their unique physiological profiles. Through a detailed comparison, we assessed key parameters such as ventilatory efficiency (VE/VCO_2_), oxygen uptake efficiency slope (OUES), oxygen uptake efficiency plateau (OUEP), and peak oxygen pulse (O_2_P_peak_) in highly trained endurance athletes. Where discrepancies were identified, we characterized these differences and created or adjusted the predictive equations to better suit the demands and capacities of endurance athletes. This tailored approach allows for more precise health assessments, performance evaluations, and the development of personalized training strategies, ensuring that endurance athletes are monitored with a higher degree of specificity and accuracy. The presented article reviews the already published outcomes of the investigation. Moreover, state-of-the-art findings regarding other CPET-obtained variables are summarized. The aim of this brief review is to (1) provide physiologists and physicians with fundamental knowledge about CPET interpretation in endurance athletes and (2) identify existing research gaps to guide further investigations regarding active and trained populations.

## 2. Methods

### 2.1. Study Design

The presented review summarizes the NOODLE study and is based on data collected during CPET carried out in the years 2022-2023 at the Institute of Sport—National Research Institute in Warsaw, Poland [27–30]. The approval from the Institutional Review Board of the Medical University of Warsaw (Pawińskiego 3C Street, 02-106 Warsaw, approval no. AKBE/277) was obtained for all the studies. Informed consent has been obtained from all the participants in this study. In total, 234 healthy members of National Teams in endurance sports (biathletes, cyclists, cross-country skiers, long-track speed skaters, middle- and long-distance runners, ski-mountaineers, and triathletes) were included in further analyses. All the participants were healthy and highly trained endurance athletes. Brief characteristics of the included participants are presented in Table 2. Well-established maximum effort criteria were fulfilled in all the included tests [30]. Depending on the study, CPET was performed on a cycloergometer as a continuous ramp test (*n* = 140) or on a treadmill as an intermittent step test (*n* = 94). All the athletes underwent CPET, and several demographic and exercise variables were analyzed to build the prediction equations. We considered the basic parameters: age, weight, height, body surface area, and body mass index, and paired them with exercise indices, e.g., VO_2_. Our approach aimed to build robust and easily available models to facilitate their usage in practical settings. All the physiological variables analyzed in the NOODLE study were VE/VCO_2_, OUES, OUEP, and O_2_P_peak_. The CPET was conducted to the maximal exertion according to the following criteria: participant declined to continue exercises with Borg scale ≥ 18, VO_2_ plateau (< 100 mL increase lasting ≥ 30 s), respiratory exchange ratio ≥ 1.05, and HRmax ≥ 80% of age-predicted. All the participants were familiar with the testing procedures and environment. CPET was performed by qualified and experienced staff, using well-established protocols, validated equipment, and manufacturers' guidelines. The detailed description of applied testing protocols may be found in the above-referenced NOODLE studies.

### 2.2. Statistical Analysis

In the first step of statistical analysis, we assessed the relationships between individual variables. We used Student's *t*-test to determine differences between different types of VE/VCO_2_: slope (interval from start to first ventilatory threshold), total (interval from start to termination), and nadir (the lowest 30 s value). We also used Student's *t*-test to compare OUES calculated from different exercise intervals: from 75%, 90%, and 100% of the duration of the exercise test. Finally, with Student's *t*-test, we compared predicted and observed values for each model.

We implemented two-way mixed effects intraclass correlation coefficient (ICC_3,1_) to confirm the agreement between predictions and direct measurements from CPET. Using ICC_3,1_, we showed whether models reflect trends and relationships among endurance athletes that occur among the general population. We also used linear regression to compare observed and predicted values, and we calculated the coefficient of determination (*R*^2^) and root mean square error (RMSE).

Then, we confirmed the choice of derivation method by analyzing data assumptions (collinearity, independence of observations, leverage plots, and autocorrelation) and aimed to generate specific models using multivariate linear regression. All the derived models and equations were subsequently validated with cross-validation.

## 3. Insights From the NOODLE Study

**Figure 1:**
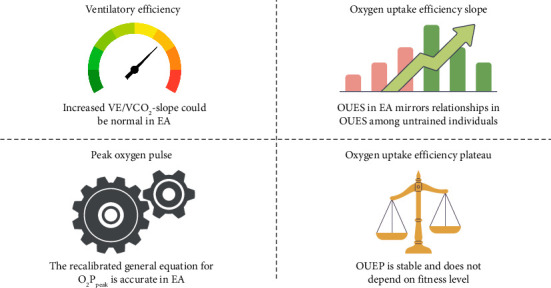
Visual summary of key findings for discussed cardiorespiratory variables among endurance athletes. Abbreviations: VE/VCO_2_-slope, ventilatory efficiency slope; EA, endurance athletes; OUES, oxygen uptake efficiency slope; OUEP, oxygen uptake efficiency plateau; O_2_P_peak_, peak oxygen pulse.

A summary of the NOODLE study outcomes is presented in Table 3 and visualized in Figure 1. More detailed explanations regarding each investigated variable will follow.

### 3.1. VE/VCO_2_

VE/VCO_2_, the ratio of pulmonary ventilation to carbon dioxide production, reflects right ventricular-pulmonary vascular function during exercise [32]. VE/VCO_2_ is most often plotted to the first ventilatory threshold (i.e., VE/VCO_2_-slope). Achieving the VE/VCO_2_-slope does not require maximum effort. The VE/VCO_2_-slope is widely used in heart failure patients, particularly those with reduced ejection fraction, predicting poor outcomes and identifying high-risk individuals during CPET [23]. In the NOODLE study, we found that the VE/VCO_2_-slope is not significantly higher in endurance athletes during physical effort compared to the untrained individuals. However, the current prediction equations do not properly reflect the relationship between demographic variables and VE/VCO_2_-slope in endurance athletes because none of the equations are exact. Additionally, we investigated the sex differences in VE/VCO_2_ measured from several intervals of physical effort. The lowest 30 s VE/VCO_2_, VE/VCO_2_-slope, and total VE/VCO_2_ were higher in female than male endurance athletes. Our findings shed new light on between-sex differences with the potential to influence training prescription regarding cardiovascular, muscular, hematologic, and pulmonary systems' interplay. As we underlined in Table 3, the new model is necessary for endurance athletes. The existing models were mostly univariable, with the inclusion of the age covariate. We observed that the impact of age on VE/VCO_2_ is negligible in the endurance athletes [29]. However, body measurements, such as height and weight, have higher prediction value in endurance athletes. Therefore, the new models for endurance athletes should revise the set of covariates and minimize the impact of age in exchange for weight and height [29].

### 3.2. OUES

OUES reflects how efficiently the body uses oxygen during exercise. Unlike other exercise test variables, OUES can be measured even during submaximal exercise, making it particularly valuable in populations who cannot push themselves to peak effort, such as elderly or frail patients [33]. This makes it a versatile tool in both athletic performance assessment and clinical care. OUES may be used for evaluating patients with cardiovascular or pulmonary conditions regarding disease prognosis and risk stratification, especially when they cannot perform maximal exercise [24, 34, 35]. In the NOODLE study, we noted that OUES is significantly higher in endurance athletes. However, despite OUES being significantly higher in well-trained individuals, it still did not differ between time intervals taken from the CPET [28]. When considering 75%, 90%, and 100% of the CPET duration, there were no significant differences. Therefore, the OUES is a more universal measurement not only in clinical populations but also in endurance athletes because it does not require performing maximal-effort CPET to objectively assess the endurance capacity [28]. The existing prediction equations in the majority correctly reflected the trends, with significant underestimation of the predictions for well-trained individuals. However, we also observed a more complex relationship between previously used covariates (age and sex) in endurance athletes than in the general population. Therefore, we derived the novel equations tailored to the needs of endurance athletes. In new models for endurance athletes, we used the interaction factor (e.g., age × sex). Finally, we recommend the consideration of interaction factors for further studies.

The updated equations for OUES from the NOODLE study were as follows [28]:

a. OUES calculated from 75% of the CPET = 1.09 + 2.87 × BSA − 0.0030 × (age × sex).
b. OUES calculated from 90% of the CPET = 1.46 − 3.02 × BSA − 0.0010 × (age × sex).
c. OUES calculated from 100% of the CPET = 1.54 − 2.99 × BSA − 0.0014 × (age × sex).

Sex = 2 for males and = 1 for females. BSA is in m^2^. Age is in years.

### 3.3. OUEP

OUEP represents a stable point where oxygen consumption becomes less responsive to increases in ventilation during exercise [36]. Clinically, it can provide valuable insights into a patient's cardiovascular and pulmonary health, offering a marker for early diagnosis of heart or lung diseases without requiring maximal exercise exertion [26, 37]. In the NOODLE study, we found that OUEP is not higher or even different in endurance athletes compared to the general population [27]. However, the prediction equations did not show satisfactory accuracy in athletes. In the NOODLE study, we did an external validation and created a new equation for OUEP. We emphasize that OUEP is a promising new understudied variable that is highly stable and does not depend on endurance capacity. We recommend OUEP to be routinely assessed during CPET in athletes, untrained individuals, and patients due to its feasibility and universal significance [27].

The updated equation for OUEP from the NOODLE study was as follows [27]:

  61.369 + 5.078 × sex − 0.123 × height.
  Sex = 1 for males and = 0 for females. Height is in cm.

### 3.4. O_2_P_peak_

O_2_P_peak_ represents the oxygen uptake per heartbeat during peak exercise, serving as a proxy for stroke volume and overall cardiopulmonary performance [38]. Clinically, it can reveal abnormalities in cardiovascular function, particularly in patients with heart and pulmonary conditions or reduced exercise tolerance [25]. In the NOODLE study, firstly, we externally validated the common prediction equation for O_2_P_peak_ derived from the “Fitness Registry and the Importance of Exercise: A National Data Base” (FRIEND database) on the cohort of endurance athletes. We observed a similar relationship as in OUES, i.e., the model significantly underestimated the O_2_P_peak_ but generally reflected the relationships between exercise variables and body measurements [30]. Hence, we proposed a new method of calibration for prediction equations. We recalibrated the most recognized equation for O_2_P_peak_ from the FRIEND database with the usage of multivariable linear regression [39]. This procedure allows us to adjust the prediction equation to any population by modifying the basic coefficients without changing the core of the model. The adjusting method that we proposed in the NOODLE study allows for the transfer of equations between populations with different characteristics [30]. Hence, it is a simpler solution than creating a completely new equation, as was recommended in previous studies.

The updated equation for O_2_P_peak_ from the NOODLE study was as follows [30]:

  24.824 − 0.0963 age − 7.062 × sex.
  Sex = 0 for males and = 1 for females. Age is in years.

## 4. Recommendations for Practitioners and Directions for Further Research

The NOODLE study exhibits limitations that should be acknowledged and addressed in further research regarding CPET. The presented study is a cross-sectional one. The longitudinal setting would be a valuable study design, allowing for exploration not only of associations, but also the influence of endurance training on the described variables. Only highly trained subjects took part in the study. Other trained populations, especially amateur endurance athletes who started training in middle or late life, and lifelong athletes, should be investigated. To date, studies on such groups remain scarce. Moreover, patients undertaking physical training simultaneously with existing conditions, especially lifestyle diseases, are understudied in the abovementioned context. Finally, near-infrared spectroscopy might be combined with CPET to identify central and peripheral exercise limitations and possibly improve diagnostic accuracy and risk stratification. This novel approach is becoming more popular in high-performance athletic settings in the context of training optimization. However, research regarding its application in clinical cardiopulmonary diagnostics remains lacking.

## 5. Other Variables

The rationale behind the NOODLE study was based on the hypothesis that general predictions and equations might not accurately reflect the unique physiological profiles of well-trained endurance athletes. As presented above, some of the CPET-obtained variables are significantly different in the general population and endurance athletes and others may be approached in a similar way in both groups. The presented research addressed the existing research gap and reported novel findings. Our work fits into the existing trend of precision medicine that tailors diagnostics, treatments, and interventions to individual patients' characteristics.

In recent years, such an approach gained noteworthy interest, and our findings should be reported in a wider context. Most importantly, the available literature suggests that other CPET-obtained indices differ between endurance athletes and the general population. Especially, predicted maximum oxygen uptake (VO_2_max) and HRmax should reach only up to 84% and > 90%, respectively, in untrained individuals according to ATS/ACCP guidelines [12]. However, simultaneously, EACPR/AHA guidelines for well-trained individuals recommend more than 100% for VO_2_max and ≥ 85% for HRmax [40]. Moreover, the breathing reserve is also different between aforementioned guidelines and should be > 15% in the general population compared to > 20% in athletes.

VO_2_max is a key indicator of aerobic fitness, cardiovascular health, and a strong mortality and longevity predictor [41]. Typically, VO_2_max is significantly higher in endurance athletes compared to other populations, although the exact difference may depend on trained sport, ethnicity, or exercise modality [42]. In healthy population, VO_2_max typically decreases with age, is higher in males compared to females, and is higher on running treadmill compared to cycloergometry [6, 43, 44]. However, the extent of these differences is not thoroughly investigated in the trained athletic population. Multiple VO_2_max prediction equations were already analyzed in recreational and elite athletes [45, 46]. The available models were characterized with moderate accuracy; therefore, predictive equations should not replace direct VO_2_max determination during CPET in endurance athletes [46, 47]. Consequently, deriving improved specific models and equations addressing both recreational and elite endurance athletes is recommended.

Monitoring HRmax provides insights into a person's exercise capacity, heart function, and overall fitness [48]. Achieving or failing to achieve the predicted HRmax can indicate the presence of cardiovascular limitations, autonomic dysfunction, or potential exercise intolerance, all of which are important in diagnosing heart disease, evaluating treatment effectiveness, or determining exercise prescription [49, 50]. In healthy population, HRmax tends to decrease with age and level of fitness [44]. Since maximal exercise testing is not feasible in many settings, HRmax is often estimated using age-predicted equations with multiple options already presented in the literature [48–51]. Typically, HRmax in trained athletes is lower compared to their sedentary counterparts [52]. Noteworthy differences in HRmax depending on exercise modality were observed in trained athletes: higher values were obtained on rowing ergometer and running treadmill compared to kayak, skiing, and cycling ergometers [53]. Generally, the larger the muscle mass involved, the higher the VO_2_max and HRmax. Compared to other parameters, HRmax may be well predicted in active and trained populations. Formulas from Tanaka et al. (202.5–0.53 × age) and (208–0.7 × age) yield acceptable accuracy and present relatively low mean errors [48, 51].

Breathing reserve reflects the capacity of the respiratory system to handle increased demand during exercise [54]. Monitoring breathing reserve during CPET is important because it helps determine if a patient's exercise limitation is due to respiratory factors. A low breathing reserve may indicate ventilatory impairment, such as in chronic obstructive pulmonary disease. In contrast, a high breathing reserve suggests that other systems, like the cardiovascular system, may be the limiting factor [54]. The analysis of breathing reserve may support identifying mechanical limits to exercise, associated with obstructive or restrictive lung disease [54]. Breathing reserve is typically lower in endurance athletes compared to the general population. In regular population, the lung capacity is rarely the limiting factor in exercise [31, 55]. However, in trained subjects, the discrepancy in adaptation between cardiovascular, pulmonary hematologic, and muscular systems exists [56]. Even long-time and demanding endurance training is not sufficient to significantly improve pulmonary mechanical systems without additional respiratory muscle training [57]. Consequently, highly trained athletes may use all available lung mechanical capacity during peak exercise and even exhibit breathing reserve corresponding to 0% [31]. The percentage of breathing reserve may also be sport-dependent, since swimmers and rowers tend to exhibit increased lung volumes and pulmonary diffusion capacity when compared to other sports [14, 58]. Overall, large variability may be observed without clear physiological explanation. Further research might explore where this variability comes from, including differences between sexes and sports disciplines.

## 6. Conclusions

The presented review summarized state-of-the-art research regarding CPET-obtained variables for endurance athletes. When necessary, adjusted reference values and predictive equations that reflect unique physiological profiles of endurance athletes were presented. As more people engage in endurance sports with limited medical oversight, tailored CPET assessments are crucial for accurate performance evaluation and health monitoring. By characterizing differences between general formulas and those suited for endurance athletes, we offered improved tools for optimizing training and ensuring athlete safety.

## Figures and Tables

**Table 1:** Comparison of reference values for peak oxygen uptake.

Variable	Age group (years)	Males				Females			
		Cycling CPET		Running CPET		Cycling CPET		Running CPET	
		FRIEND [6]	CHEER [5]	FRIEND [6]	CHEER [5]	FRIEND [6]	CHEER [5]	FRIEND [6]	CHEER [5]
VO_2_peak (mL/kg/min)	Younger EA	44.2 ± 12.4	56.7 ± 9.6	44.7 ± 11.5	62.0 ± 11.1	32.0 ± 10.1	44.7 ± 7.2	35.3 ± 9.7	52.0 ± 8.6
	Older EA	31.6 ± 11.1	52.1 ± 8.1	39.0 ± 11.4	52.8 ± 7.6	22.8 ± 7.7	37.8 ± 4.4	29.3 ± 8.6	46.9 ± 6.7

*Note:* Table summarizes data for individuals aged 18–45 to most exactly match the age of participants from the NOODLE study. FRIEND registry is the most recognized and widely used data source of reference values for CPET among general population. CHEER registry is the most recognized and widely used data source of reference values for CPET among endurance athletes. Data from FRIEND registry used the RER of 1.1 and data from CHEER registry used RER of 1.05. Younger EA were 18–30 years old in CHEER database and 20–29 years old in FRIEND database. Older EA were 30–45 years old in CHEER database and 30–39 in FRIEND database.

Abbreviations: CHEER, Cardiopulmonary Health and Endurance Exercise Registry; CPET, cardiopulmonary exercise testing; EA, endurance athletes; FRIEND, Fitness Registry and the Importance of Exercise: A National Data Base; VO_2_peak, peak oxygen uptake.

**Table 2:** Participants' characteristics.

Variable	Type of CPET			
	Running (*N* = 94)		Cycling (*N* = 140)	
	Males (*N* = 62, 66.0%)	Females (*N* = 32, 34.0%)	Males (*N* = 77, 55.0%)	Females (*N* = 63, 45.0%)
Age (years)	28.1 ± 5.5	26.4 ± 5.0	21.8 ± 4.8	23.8 ± 4.2
Weight (kg)	76.1 ± 10.2	60.6 ± 8.3	76.1 ± 7.6	61.0 ± 5.5
Height (cm)	181.2 ± 7.3	169.5 ± 7.9	181.6 ± 6.3	166.3 ± 6.2
BMI (kg/m^2^)	23.2 ± 2.5	21.0 ± 1.8	23.1 ± 1.7	22.1 ± 1.6
BSA (m^2^)	2.0 ± 0.2	1.7 ± 0.2	2.0 ± 0.2	1.7 ± 0.1
VO_2_max (mL/kg/min)	59.2 ± 0.6	51.9 ± 6.3	57.8 ± 9.0	52.1 ± 7.0

*Note:* Data are presented as mean (standard deviation).

Abbreviations: BMI, body mass index; BSA, body surface area; CPET, cardiopulmonary exercise testing; VO_2_max, maximal oxygen uptake.

**Table 3:** A brief summary of the NOODLE study outcomes.

Variable	Athlete-specific data		Was it different between the general population and endurance athletes?	Statistical comparison between general population and endurance athletes	*R* ^2^ for general models applied to endurance athletes	Direction of differences in endurance athletes	Degree of change between the general population and endurance athletes	Solution proposed in NOODLE study
	Males	Females						
VE/VCO_2_-slope [29]	26.1 ± 2.0	27.7 ± 2.6	Yes	ICC =< 0.001–0.44 *R*^2^ = 0.003–0.031	0.003–0.031	Higher	↑	New model

OUES (mL/min/L/min) [28]	OUES_75_ = 4.53 ± 0.90 OUES_90_ = 4.52 ± 0.91 OUES_100_ = 4.41 ± 0.87	OUES_75_ = 3.50 ± 0.65 OUES_90_ = 3.49 ± 0.62 OUES_100_ = 3.41 ± 0.58	Yes	ICC = 0.062–0.529 *R*^2^ = 0.004–0.388	0.004–0.324	Higher	↑↑	New model

OUEP (mL/L) [27]	44.2 ± 4.2	41.0 ± 4.8	No	Males: *t* = −0.86, *p*=0.39 Females: *t* = 0.54, *p*=0.59	0.099	Comparable	⟶	—

O_2_P_peak_ (mL/beat) [30]	23.6 ± 2.8	16.4 ± 2.0	Yes	Males: *t* = − 8.06, *p* < 0.001 Females: *t* = −6.06, *p* < 0.001	0.62	Higher	↑↑	Recalibrated equation

*Note:* The definitions of “moderately” and “significantly” higher were adapted based on Petek et al. [31]. *R*^2^, adjusted coefficient of determination. Differences for OUES and VE/VCO_2_-slope were conducted with the usage of ICC (intraclass correlation coefficient) and *R*^2^. Differences between general population and untrained individuals for OUEP and O_2_P_peak_ were analyzed with the usage of Student's *t*-test.

Abbreviations: O_2_P_peak_, peak oxygen pulse; OUES, oxygen uptake efficiency slope; OUES_75_, oxygen uptake efficiency slope calculated from 75% of the duration of exercise test; OUES_90_, oxygen uptake efficiency slope calculated from 90% of the duration of exercise test; OUES_100_, oxygen uptake efficiency slope calculated from 100% of the duration of exercise test; OUEP, oxygen uptake efficiency plateau; VE/VCO_2_, ventilatory efficiency.

↑, variable moderately higher in endurance athletes than in untrained population.

↑↑, variable significantly higher in endurance athletes than in untrained population.

⟶, variable comparable between endurance athletes and general population.

## Data Availability

Data sharing is not applicable to this article as no new data were created or analyzed in this study.
